# Association between high-density mapping of atypical atrial flutter, clinical outcomes and healthcare utilization

**DOI:** 10.1111/jce.16355

**Published:** 2024-07-04

**Authors:** Joshua Sink, Kasen Culler, Lakshmi Uppalapati, Nicola Lancki, Graham Peigh, Graham Lohrmann, Mahmoud Elsayed, Herman Carneiro, Jayson Baman, Anna Pfenniger, Kaustubha D. Patil, Nishant Verma, Rishi Arora, Susan S. Kim, Alexandru B. Chicos, Albert C. Lin, Bradley P. Knight, Rod S. Passman

**Affiliations:** 1 Department of Internal Medicine, Northwestern University, Chicago, Illinois, USA; 2 Department of Internal Medicine, Baylor University, Houston, Texas, USA; 3 Department of Preventative Medicine, Northwestern University, Chicago, Illinois, USA; 4 Division of Cardiology, Northwestern University, Chicago, Illinois, USA

**Keywords:** atypical atrial flutter, healthcare utilization, high density mapping

## Abstract

**Background:**

Success of atypical atrial flutter (AAFL) ablation has historically been limited by difficulty mapping the complex re-entrant circuits involved. While high-density (HD) mapping has become commonplace in clinical practice, there are limited data on outcomes of HD versus non-HD mapping for AAFL ablation.

**Objective:**

To compare clinical outcomes and healthcare utilization using HD mapping versus non-HD mapping for AAFL ablation.

**Methods:**

Retrospective analysis of all AAFL procedures between 2005 and 2022 at an academic medical center was conducted. Procedures utilizing a 16-electrode HD Grid catheter and Precision mapping system were compared to procedures using prior generation 10–20 electrode spiral catheters and the Velocity system (Abbott, IL). Cox regression models and Poisson regression models were utilized to examine procedural and healthcare utilization outcomes. Models were adjusted for left ventricular ejection fraction, CHA_2_DS_2_-VASc, and history of prior ablation.

**Results:**

There were 108 patients (62% HD mapping) included in the analysis. Baseline clinical characteristics were similar between groups. Use of HD mapping was associated with a higher rate of AAFL circuit delineation (92.5% vs. 76%; *p* = .014) and a greater adjusted procedure success rate, defined as non-inducibility at procedure end, (aRR (95% CI) 1.26 (1.02–1.55) *p* = .035) than non-HD mapping. HD mapping was also associated with a lower rate of ED visits (aIRR (95% CI) 0.32 (0.14–0.71); *p* = .007) and hospitalizations (aIRR (95% CI) 0.32 (0.14–0.68); *p* = .004) for AF/AFL/HF through 1 year. While there was a lower rate of recurrent AFL through 1 year among HD mapping cases (aHR (95% CI) 0.60 (0.31–1.16) *p* = .13), statistical significance was not met likely due to the low sample size and higher rate of ambulatory rhythm monitoring in the HD group (61% vs. 39%, *p* = .025).

**Conclusion:**

Compared to non-HD mapping, AAFL ablation with HD mapping is associated with improvements in the ability to define the AAFL circuit, greater procedural success, and a reduction in the number of ED visits and hospitalization for AF/AFL/HF.

## 1 | BACKGROUND

Atrial flutter (AFL) is the second most common arrhythmia in the United States, with rapidly increasing prevalence nationwide.^1,2^ The development of AAFL, particularly left-sided AAFL, is often associated with prior atrial fibrillation ablation via pulmonary vein isolation or cardiac surgery.^3^ Specifically, AAFL circuits necessitate areas of slow conduction, which are often a result of prior procedures, for propagation. While prior studies demonstrate that AAFL circuits most commonly depend on the mitral isthmus or left atrial roof, comprehensive localization is necessary before ablation attempts.^1,4,5^

High density (HD) electroanatomic mapping systems allow for thousands of myocardial points to be mapped for voltage and activation in a short period of time. Indeed, prior studies have shown that the use of an HD mapping catheter results in shorter mapping times and more selective identification of low voltage areas than the previous generation mapping catheters.^6^ In studies examining atrial fibrillation ablation, use of an HD mapping catheter has been associated with improved freedom from atrial arrhythmias.^7^ To date, there are limited data on the association between HD mapping and AAFL ablation outcomes.

The objective of the present study is to assess the associations between HD mapping during AAFL ablation, clinical outcomes following ablation, and subsequent healthcare utilization (HCU).

## 2 | METHODS

### 2.1 | Study design and population

This retrospective study reviewed all patients who underwent AAFL ablation at a single quaternary academic medical center between 2005 and 2022. Inclusion criteria were AAFL ablation procedures using Ensite or NavX mapping (Abbott, IL). Patients were separated into two groups: (1) those who had the procedure performed utilizing the HD grid mapping catheter and Precision mapping system (Abbott, IL), and (2) patients who had the procedure with traditional spiral mapping catheters and the “low density” Velocity mapping system (Abbott, IL). Procedures in group 1 occurred between 2018 and 2022. Procedures in group 2 occurred between 2005 and 2018. Baseline characteristics, procedural characteristics, procedural outcomes, clinical outcomes, and healthcare utilization (HCU) through 1 year of follow-up were recorded and compared between groups. The study was approved by the Institutional Review Board at Northwestern University.

### 2.2 | Procedural workflow

AAFL mapping and ablation was performed using standardized technique. AAFL was diagnosed via a comprehensive electrophysiology study, including entrainment maneuvers. When AAFL was determined to originate in the left atrium, transeptal access was pursued under intracardiac echocardiographic and fluoroscopic guidance.

For the low-density mapping group, electroanatomic mapping was performed via a spiral mapping catheter (Reflexion or AFocus models, Abbott, IL) and the Velocity mapping system. These mapping catheters included 10–20 electrodes, with between 2 and 7 mm electrode spacing depending on the specific model, which allowed for 5–10 bipolar signals to be saved per beat. The Velocity software system required manual point collection. Ablation was performed with either aTacticath^™^ contact force sensing catheter or a non-force sensing irrigated 4 mm ablation catheter.

In the HD mapping group, electroanatomic mapping of the atria was performed utilizing the omnipolar HD grid mapping catheter and Precision mapping system. This catheter features 16 electrodes organized in a 4 × 4 pattern. Electrode size is 1 mm and spacing is 3 mm. The 4 × 4 orthogonal grid design allows bipolar signals to be recorded not only parallel, but also perpendicular to the spline, which is unique from traditional spiral catheters. This allows for 36 bipolar signals to be saved per beat. The Precision software system introduced “auto-mapping,” which is capable of automatic point collection for every beat within pre-defined parameters. Ablation was performed with a Tacticath contact force sensing catheter (Abbott, IL).

Propagation and voltage mapping were performed in AFL in all cases. If the patient arrived in sinus rhythm, atrial burst pacing was performed to induce AFL. In all cases, the critical isthmus was targeted for ablation. If ablation was performed along the posterior left atrium, esophageal temperature was monitored.

After ablation, an electrophysiology study was performed, and burst pacing was attempted to re-induce the flutter. Isoproterenol was often used when attempting to re-induce AFL. The procedure was considered successful if the rhythm was non-inducible after ablation.

### 2.3 | Follow-up

Recurrent arrhythmia was evaluated using ECGs, Holter monitors, and implantable cardiac devices, when present, at 3-, 6-, and 12-months postprocedure, and in response to suggestive symptoms. The first 3 months were considered a blanking period for recurrence. Electronic medical records were reviewed to adjudicate ablation-related adverse events and HCU metrics. The Care Everywhere tool in Epic (Verona, WI) was used to help capture clinical events outside of the primary medical center.

### 2.4 | Outcomes

The primary outcomes evaluated were procedure success and AFL recurrence rates. Secondary outcomes included HCU at 30d and 1 y following ablation, adverse events within 30 days of ablation, successful circuit delineation, need for repeat procedure, and major adverse cardiovascular events. Successful circuit delineation was classified based on review of procedural reports and electroanatomic maps and was defined by clear identification of the entire cycle length propagation and critical isthmus.

### 2.5 | Statistical analysis

Data are summarized as mean ± standard deviations, medians [IQR], or counts (%). Comparisons between the groups were based on two-sample *t*-tests with equal variances, Wilcoxon’s rank sum test, chi-square, or Fisher’s exact test, as appropriate.

Cox regression models were used to evaluate the association of HD mapping on time to event outcomes of AFL recurrence and death. To evaluate the association of HD mapping on the outcomes of procedure success, redo procedure, adverse events within 30 days, ED visits within 30 days, and readmissions within 30 days, cardioversions, and any ED or hospitalizations for AF/AFL/HF, modified Poisson regression models with robust error variances were used. To evaluate the association of HD mapping on number of hospitalizations for AF/AFL/HF within a year, number of ED visits for AF/AFL/HF within a year, Poisson regression models for count outcomes were used. To account for some patients not having a full year of follow-up, an offset term (log of follow-up time), was added to the Poisson model. Univariable and multivariable models were run. All multivariable models were adjusted for selected potential confounders including left ventricular ejection fraction, CHA_2_DS_2_-VASc, and history of prior ablation. *P* values < .05 were considered statistically significant.

## 3 | RESULTS

### 3.1 | Baseline characteristics

A total of 108 patients (65 ± 10 years old, 30% female) were included in this analysis with 67 patients (62%) in the HD group and 41 patients (38%) in the non-HD group. The HD group had a higher percentage of females. Baseline characteristics were otherwise similar between groups (Table 1). There was no difference in postprocedure antiarrhythmic or anticoagulant drug use between groups. Use of contact force was significantly higher in the HD group (100% vs. 37%, *p* < .001). There was a higher rate of ambulatory rhythm monitoring in the HD group (61% vs. 39%, *p* = .025).

### 3.2 | Procedural characteristics and clinical outcomes

**FIGURE 1:**
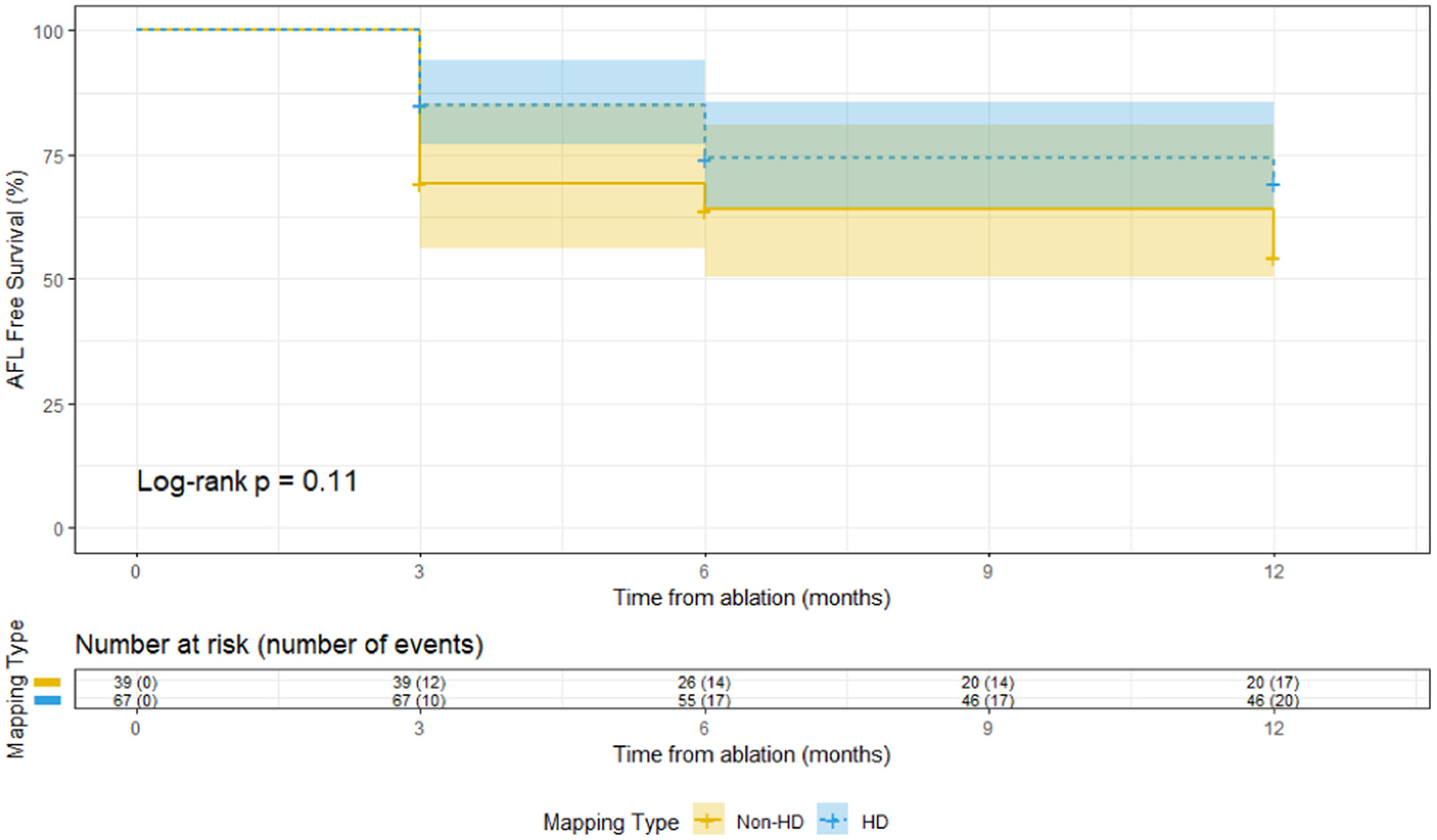
Time to recurrence of atrial flutter (AFL) through 1 year of high-density (HD) group versus non-HD group.

The distribution of AFL location and chamber was similar between the cohorts (Table 2). The majority of AAFLs were mitral or LA roof dependent. Use of HD mapping was associated with a higher rate of AAFL circuit delineation (92.5% vs. 76%; *p* = .014). The rate of procedure success was higher in the HD group (Table 3; 91% vs. 71%, *p* = .006). There were no significant differences in the number of adverse events postprocedure, or all-cause ED visits or readmissions within 30 days between groups (Table 4). After adjustment for left ventricular ejection fraction, CHA_2_DS_2_-VASC, and prior ablation, use of HD mapping remained associated with greater procedure success rates (Table 5; aRR 1.26, 95% CI 1.02–1.55, *p* = .035). While there was a lower rate of recurrent AFL through 1 year among HD mapping cases (aHR 0.60 (0.31–1.16) *p* = .13), statistical significance was not met (Figure 1). Incidence of new onset of heart failure, stroke, or death was also comparable between cohorts (Table 3).

### 3.3 | Healthcare utilization

Patients in the HD group had a lower number of unadjusted ED visits and hospitalization for AF/AFL/HF through 1 year than those in the non-HD group (Table 4). Twelve percentage of patients had one or more ED visits in the HD group as compared to 29% in the non-HD group (*p* = .036). 13% of patients had one or more hospitalizations in the HD group as compared to 27% in the non-HD group (*p* = .19). After adjustment for confounders, HD mapping was associated with a lower number of ED visits (aIRR 0.32 (0.14–0.71); *p* = .007) and hospitalizations (aIRR 0.32 (0.14–0.68); *p* = .004) for AF/AFL/HF through 1 year (Table 5).

### 3.4 | Contact force subgroup analysis

Contact force was utilized in 67 (100%) patients in the HD group and 15 (37%) patients in the non-HD group. Within this subgroup, (Table 6) the HD group had a higher rate of procedure success (91% vs. 67%, *p* = .025). There was no significant difference between AFL recurrence, ED visits, or hospitalizations through 1 year (Table 6).

## 4 | DISCUSSION

In this study, we found that HD mapping of AAFL circuits was associated with greater procedure success and a reduction in the number in ED visits and hospitalization for AF/AFL/HF through 1 year of follow-up after adjusting for potential confounders. While high-density (HD) mapping has become commonplace in clinical practice, these data support its use in AAFL ablation.

HD mapping technology allows for rapid and efficient electro-anatomical map creation with high-resolution visualization of substrate. AAFL ablation stands to improve significantly from these technologies due to the complex re-entrant circuits involved. However, to date, there has been limited evaluation of the clinical benefit of HD mapping on AAFL ablation. In this study, we compared the HD grid catheter and Precision software system against prior generation technology. The orthogonal design of the HD grid generates 36 unique bipolar signals to be saved per beat, and Precision software was the first NavX system to introduce “auto-mapping,” which allowed automatic point collection for every beat within pre-defined mapping parameters. These updates produced a several-fold increase in point-collection density and speed, providing higher-resolution visualization of the substrate.^6,7^ More recently, software upgrades have introduced omnipolar signal processing which allows for wavefront visualization from single depolarizations.^8^ Omnipolar mapping can provide information independent of a specific reference time, helping to overcome common procedural roadblocks of an unstable reference electrode or variable cycle length.^8^ This has the potential for improved mapping compared to conventional bipolar local activation time (LAT) techniques.

In line with the results observed from the current study, other single center analyses have demonstrated higher rates of procedure success and lower rates of AFL recurrence through 1 year when compared against traditional mapping techniques.^9,10^ Raymond-Paquin et al.^10^ conducted a study of 62 patients who underwent atypical AFL ablation utilizing HD grid catheter mapping and found that 65% of patients remained free of any atrial tachycardia at an average of 14 months. Additionally, Balt et al.^11^ conducted a study of 82 patients undergoing atypical AFL ablation which demonstrated that use of the HD Grid catheter in combination with the Ensite Precision mapping software resulted in higher rates of acute procedural success and AFL-free survival at 1 year when compared with traditional low-density mapping. The present study expands on these data by assessing the association of high-density mapping of AAFL on downstream healthcare utilization. Notably, we found that high density mapping with the HD grid catheter and Ensite Precision mapping system was associated with lower rates of ED visits and hospitalizations when compared against traditional mapping techniques. Specifically, there was a more than two-thirds reduction in rates of ED visits and hospitalizations for AF/AFL/HF through 1 year.

The explanation for this significant difference in clinical outcomes likely originates from the ability to properly delineate the circuit in a higher percentage of cases with HD mapping. In our study, HD mapping led to successful circuit delineation in 92.5% of patients, as compared to only 76% of non-HD cases. The enhanced ability to delineate the AAFL circuit was reflected in superior procedure success amongst the HD group (91% vs. 71%). The procedural success rate observed in the current study is slightly superior to that of prior studies of HD mapping for AAFL ablation which have cited success rates of 78%–80%.^10,12^

While there was a trend towards lower rate of recurrent AFL through 1 year among HD mapping cases, the small sample size may have limited the ability to reach statistical significance. Indeed, the 40% reduction in risk of AFL recurrence with HD mapping is numerically greater than that of prior studies that have demonstrated a statistically significant reduction in recurrent AFL when HD mapping is utilized.^11^ It is likely that differential rates of ambulatory rhythm monitoring impacted diagnosis of recurrent arrhythmia. As the likelihood of diagnosing an arrhythmia is directly proportional to the time of monitoring, it is possible that the increased frequency of ambulatory rhythm monitoring in the HD group led to a higher frequency of arrhythmia detection.^13^ Thus, differences in ambulatory monitoring and/or AFL burden may explain why the difference in AFL recurrence was not great enough to reach statistical significance, and yet a significant difference in healthcare utilization was still detected.

## 5 | LIMITATIONS

Our study is limited as a single center, non-randomized, retrospective study with a relatively small sample size. It is also limited by the span of years used for procedure inclusion, as operators’ skill level likely rose over time. It is possible that variable index ablation strategies over time had an impact on the substrate for AAFL, however we would not expect this to substantially impact the primary outcomes of this study. There was a higher rate of contact force ablation catheter use in the HD group, which may have contributed to differences in outcomes. A future multi-center study powered to adjust for contact force would be helpful to answer this. Furthermore, the current study assesses a single manufacturer’s catheters and mapping systems to reduce the impact of heterogeneity between vendors. Future studies may assess the association between HD mapping and AAFL outcomes using catheters and software from other vendors. Total mapping time and ablation time were additional variables that may be associated with use of HD mapping. Unfortunately, these variables were not routinely documented in procedure notes and therefore could not be analyzed in the present study. Similarly, the total number of mapping points was not routinely documented and therefore could not be compared between groups.

## 6 | CONCLUSION

Compared to non-HD mapping, AAFL ablation with HD mapping is associated with improvements in the ability to define the AAFL circuit, greater procedural success, and a reduction in the number in ED visits and hospitalization for AF/AFL/HF. Further large-scale analyses are necessary to determine the association between procedural success and long term AAFL recurrence.

## Figures and Tables

**TABLE 1:** Baseline characteristics.^a^

Characteristic	Non-HD (*N* = 41)	HD (*N* = 67)	*p*-value
Mean Age ± SD	64 ±10	66 ±10	.26
Female	6 (15%)	26 (39%)	.008
Mean CHA2DS2-VASc ± SD	2.29 ± 1.63	2.62 ± 1.64	.30
CHF	12 (29%)	14 (21%)	.34
CAD	13 (32%)	13 (20%)	.16
CVA	6 (15%)	11 (17%)	.78
DM II	8 (20%)	10 (15%)	.56
Class I AAD Use	8 (20%)	11 (16%)	.68
Class II AAD Use	25 (61%)	41 (61%)	.98
Class III AAD Use	24 (59%)	32 (48%)	.28
Class IV AAD Use	9 (22%)	12 (18%)	.61
Anticoagulant Use	38 (93%)	64 (96%)	.67
Mean LVEF ±SD	52 ±12	53 ±10	.89
Dilated LA	31 (84%)	45 (74%)	.25
Prior PVI	29 (71%)	51 (76%)	.54
Prior MAZE	6 (15%)	17 (25%)	.19

*Note*: The denominator used in calculating percentages is reflective of any instances of missing patient data.

a AAD, antiarrhythmic drug; CHF, congestive heart failure; CAD, coronary artery disease; CVA, cerebrovascular accident; DM II, diabetes mellitus II; LVEF, left ventricle ejection fraction; LA, left atrium; PVI, pulmonary vein isolation.

**TABLE 2:** Procedure characteristics.^a^

Characteristic	Non-HD (*N* = 41)	HD (*N* = 67)	*p*-value
Number of Flutters			.44
1	29 (71%)	41 (61%)	
2	10 (24%)	18 (27%)	
3+	2 (4.9%)	8 (12%)	
Side			.16
Left	40 (98%)	58 (87%)	
Right	1 (2.4%)	5 (7.5%)	
Both	0 (0%)	4 (6.0%)	
Location: LA Macro	0 (0%)	1 (1.5%)	>.99
Location: LA Micro	4 (9.8%)	7 (10%)	>.99
Location: Mitral	17 (41%)	40 (60%)	.065
Location: Roof	10 (24%)	17 (25%)	.91
Location: RA Macro	1 (2.4%)	7 (10%)	.15
Location: Unable to Determine	10 (24%)	5 (7.5%)	.014
Mean Ablation Duration (min) ± SD	36 ±24	30 ±21	.23
Contact force Ablation Catheter Use	15 (37%)	67 (100%)	<.01
Follow-up with ECG	39 (95%)	64 (96%)	>.99
Follow-up with serial ECG’s at 3, 6, and 12 months	29 (71%)	54 (81%)	.25
Follow-up with Holter	16 (39%)	41 (61%)	.025
Follow-up with PPM	11 (27%)	18 (27%)	>.99

a LA, left atrium; RA, Right atrium.

**TABLE 3:** Clinical outcomes.

Outcome	Non-HD (*N* = 41)	HD (*N* = 67)	*p*-value
Recurrence at 3 months	12 (31%)	10 (15%)	.052
Recurrence within 6 months	14 (38%)	17 (30%)	.45
Recurrence within 1 year	17 (44%)	20 (30%)	.15
Procedure Success	29 (71%)	61 (91%)	.006
Adverse Events within 30 days	5 (12%)	6 (9.0%)	.74
New onset HF	1 (2.4%)	0 (0%)	.38
Stroke	0 (0%)	4 (6.0%)	.30
Died	3 (7.3%)	4 (6.0%)	>.99

**TABLE 4:** Healthcare utilization

Utilization outcome	Non-HD (*N* = 41)	HD (*N* = 67)	*p*-value
All-cause ED within 30 days	7 (17%)	4 (6.0%)	.10
Readmission within 30 days	6 (15%)	3 (4.5%)	.080
Redo procedure performed	10 (24%)	10 (15%)	.22
Any Cardioversions in 1 year follow-up	8 (20%)	15 (22%)	.72
# AFL/AF/HF ED visits within 1 year			.036
0	29 (71%)	59 (88%)	
1	10 (24%)	7 (10%)	
2	0 (0%)	1 (1.5%)	
3	1 (2.4%)	0 (0%)	
4	1 (2.4%)	0 (0%)	
# AFL/AF/HF hospitalizations within 1year			.19
0	30 (73%)	58 (87%)	
1	7 (17%)	8 (12%)	
2	2 (4.9%)	1 (1.5%)	
3	1 (2.4%)	0 (0%)	
4	1 (2.4%)	0 (0%)	

**TABLE 5:** Regression analysis of utilization of high-density (HD) versus non-HD.^a^

	Unadjusted			Adjusted		
Outcome	HR	95% CI	*p*-value	HR	95% CI	*p*-value
AFL Recurrence within 1 year	0.59	0.31, 1.13	.11	0.6	0.31, 1.16	.13
Risk of Death	2.58	0.43, 15.4	.3	2.71	0.43, 17.0	.29
Outcome	RR	95% CI	*p*-value	RR	95% CI	*p*-value
Procedure Success	1.29	1.04, 1.59	.019	1.26	1.02, 1.55	.035
Redo Procedure	0.61	0.28, 1.34	.22	0.54	0.23, 1.27	.16
Adverse Events in 30 days	0.73	0.24, 2.25	.59	0.63	0.24, 1.67	.35
Cardioversion within 1year	1.06	0.49, 2.29	.88	1.1	0.50, 2.40	.82
All-cause ED visits within 30 days	0.35	0.11, 1.12	.077	0.27	0.08, 0.92	.037
Readmission within 30 days	0.31	0.08, 1.16	.081	0.31	0.09, 1.10	.07
Outcome	IRR	95% CI	*p*-value	IRR	95% CI	*p*-value
Number of AFL/AF/HF ED Visits in 1 year	0.30	0.13, 0.66	.003	0.32	0.14, 0.71	.007
Number of AFL/AF/HF Hospitalizations in 1 year	0.31	0.14, 0.67	.003	0.32	0.14, 0.68	.004

a HR, Hazard ratio; CI, Confidence Interval; RR, Relative Risk; IRR, Incidence Rate Ratio.

**TABLE 6:** Contact force subgroup.

Outcome	Non-HD (*N* = 15)	HD (*N* = 67)	*p*-value
Procedure success	10 (67%)	61 (91%)	.025
Any Recurrence within 1 year	4 (29%)	20 (30%)	>.99
Any ED Visits for AFL within 1 year	0 (0%)	3 (4.5%)	>.99
Any Hospitalizations for AFL within 1 year	0 (0%)	4 (6.0%)	>.99

## Data Availability

The data that support the findings of this study are available from the corresponding author upon reasonable request.
